# Supplementary material for: Polycaprolactone Composite Micro/Nanofibrous Material as an Alternative to Restricted Access Media for Direct Extraction and Separation of Non-Steroidal Anti-Inflammatory Drugs from Human Serum Using Column-Switching Chromatography
Source: Nanomaterials (Basel). 2021 Oct 12;11(10):2669. doi: 10.3390/nano11102669 (PMC8540724; doi:10.3390/nano11102669)
Supplement: Supplementary file 1 [file nanomaterials-11-02669-s001.zip › nanomaterials-1387921-Supplementary Materials.pdf]

nanomaterials

Supplementary material

Polycaprolactone Composite Micro/Nanofibrous Material as an Alternative to Restricted Access Media for Direct Extraction and Separation of Non-Steroidal Anti-Inflammatory Drugs from Human Serum Using Column-Switching Chromatography

Hedvika Raabová [1], Lucie Chocholoušová Havlíková [1], Jakub Erben [2], Jiří Chvojka [2], František Švec [1] and Dalibor Šatínský [1,*]

[1] Department of Analytical Chemistry, Faculty of Pharmacy, The Charles University, Akademika Heyrovského 1203, 50005 Hradec Králové, Czech Republic; raabovah@faf.cuni.cz (H.R.); havlikova@faf.cuni.cz (L.C.H.); svecfr@faf.cuni.cz (F.Š.)

[2] Department of Nonwovens and Nanofibrous Materials, Faculty of Textile Engineering, The Technical University of Liberec, Studentská 1402/2, 46001 Liberec 1, Czech Republic; erben.jaakub@gmail.com (J.E.); jiri.chvojka@tul.cz (J.C.)

* Correspondence: satinsky@faf.cuni.cz; Tel.: +420–495–067–228

1. FIBER FABRICATION

1.1. Principle

The on-line extractions allow faster and less laborious sample treatment which contributes to their popularity. If these procedures are carried out in column-switching chromatography system, especially high mechanical stability of nanofibrous sorbents is required. The size of inter-fiber spaces (porosity of a sorbent) influencing the stability and continuity of mobile phase flow have to be considered. The porosity and mechanical stability are partially given by the fabrication procedure. The combination of meltblown and electrospinning technologies leads to a novel cotton-like material combining high-pressure resistance and high sorption capacity. Meltblown fibers with higher diameters ensure mechanical stability whereas a high surface area to volume ratio of fine electrospun nanofibers results in promising extraction efficiency. The production equipment (depicted in Figure. S1) consists of both electrospinning and meltblown systems.

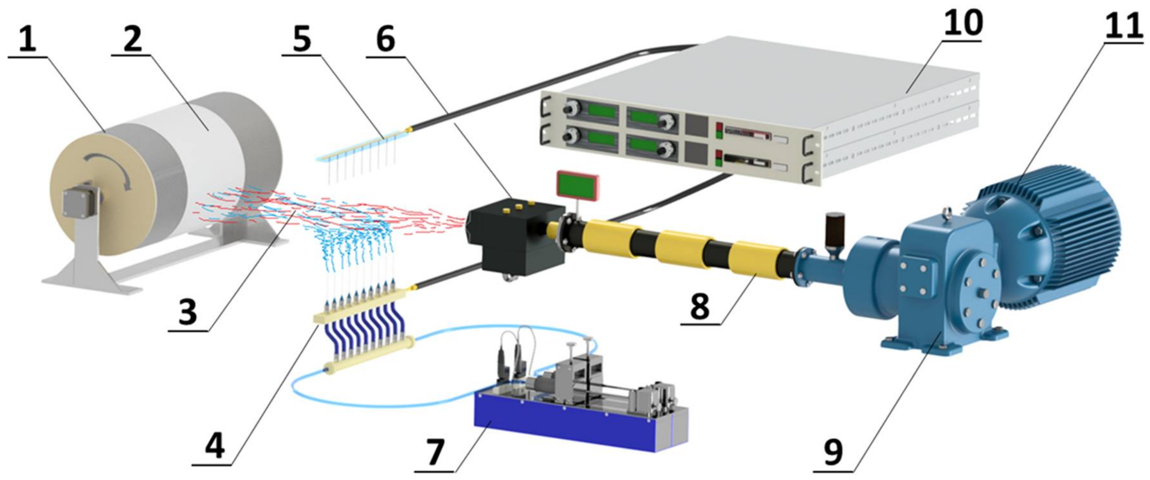

Figure S1. The combination of meltblown and electrospinning: 1—drum collector, 2—composite mi-cro-nanofiber layer, 3—airstream with fibers, 4—multi needle spinner, 5—needle counter elec-trode, 6—meltblown die, 7—pumps, 8—extruder, 9—transmission, 10—high voltage sources, 11—engine.

1.2. Fabrication

The PCL was processed by meltblown device (laboratory equipment J&M Laboratories, Lakefield Drive Duluth, USA) and passed through the die (105 orifices with 0.4 mm in diameter on 100 mm width). The extruder screw rotated 7 rpm with a flow rate 100 g/Hr. Meltblown die was heated at 190°C. Extruded melt was stretched to fibrous form using a stream of hot air at a speed of 20 ms^{-1}. PCL solution at a concentration of 16 wt% in chloroform/ethanol (9:1) was used for the electrospinning. The needle spinner containing 10 needles with a diameter of 1.2 mm with a spacing of 25 mm was used. Polymer dosage was 70 mL. h^{-1}. The spinner was charged to 35 kV and the counter electrode to -20 kV. Electrospun nanofibers combined with melted microfibers formed a stable composite material that was deposited on drum collector placed 600 mm from the meltblown die. A temperature of 23°C and a relative humidity of 45% were set during the fabrication procedure.

1.3. Morphology

Scanning electron microscopy (SEM) was used for the detection of the micro/nanofibrous material morphology. Cut discs were sputtered with a layer of gold. SEM images was taken with the VEGA3 SBU–EasyProbe microscope, Tescan (Brno, Czech Republic). The structures are shown in the following Figure S2. In the Figure S2, the combination of microfibrous scaffold covered by electrospun nanofibers can be recognized.

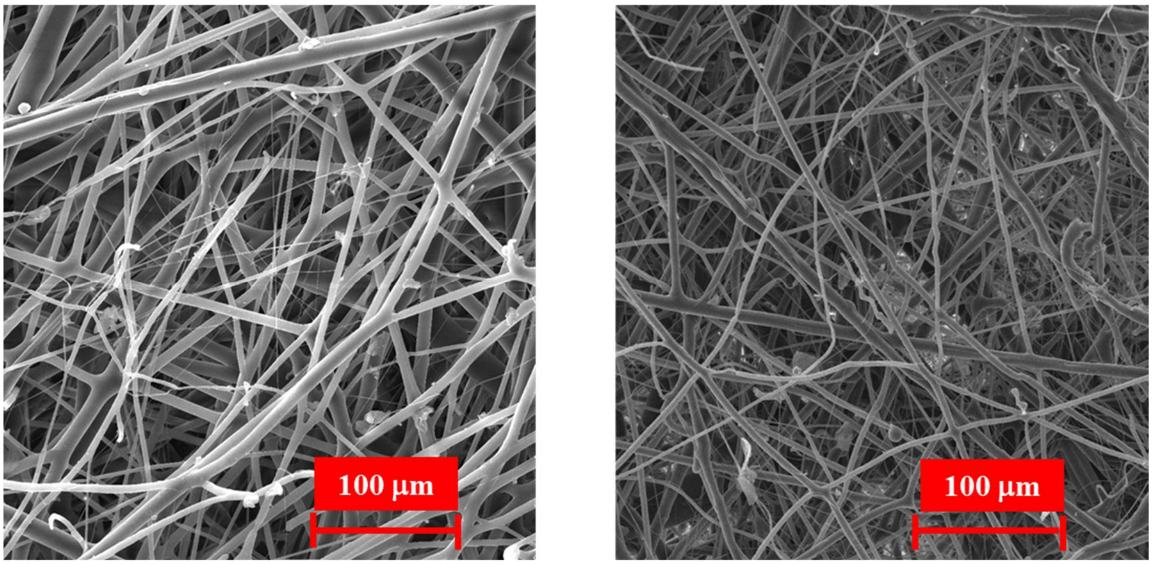

Figure S2. The morphology of micro/nanofibrous PCL material before and after using in high pressure chromatography system.

2. CARTRIDGE PREPARATION

To achieve efficient analyte extraction, the cartridge must be filled with nanofibers completely. The advantage of the composite material is that it adapts well to the shape of the cartridge and fills its space without void volumes. In our study, a commercially available PEEK cartridge was filled with a fabricated micro/nano composite material (Figure S3). The amount of the micro/nanofibrous sorbent is one of the most crucial factors to achieve high and repeatable recovery of the analytes. Using a glass rod, 44 mg of the sorbent was pressed into the cartridge. In case, there would be a need to replace the column during the experiment, the weight of the fibers should be the same to achieve repeatable results.

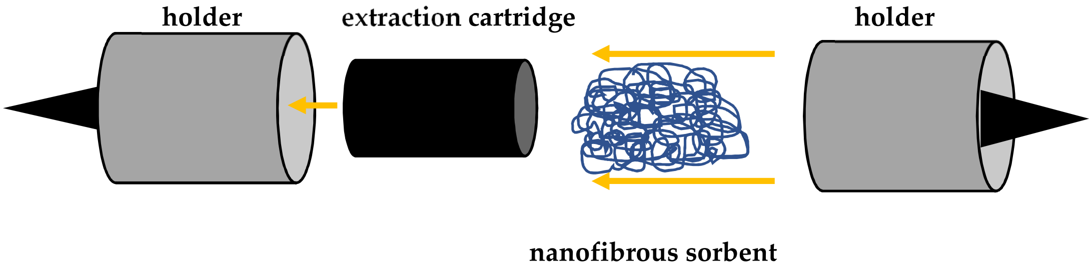

Figure S3. The filling process of the extraction cartridge.

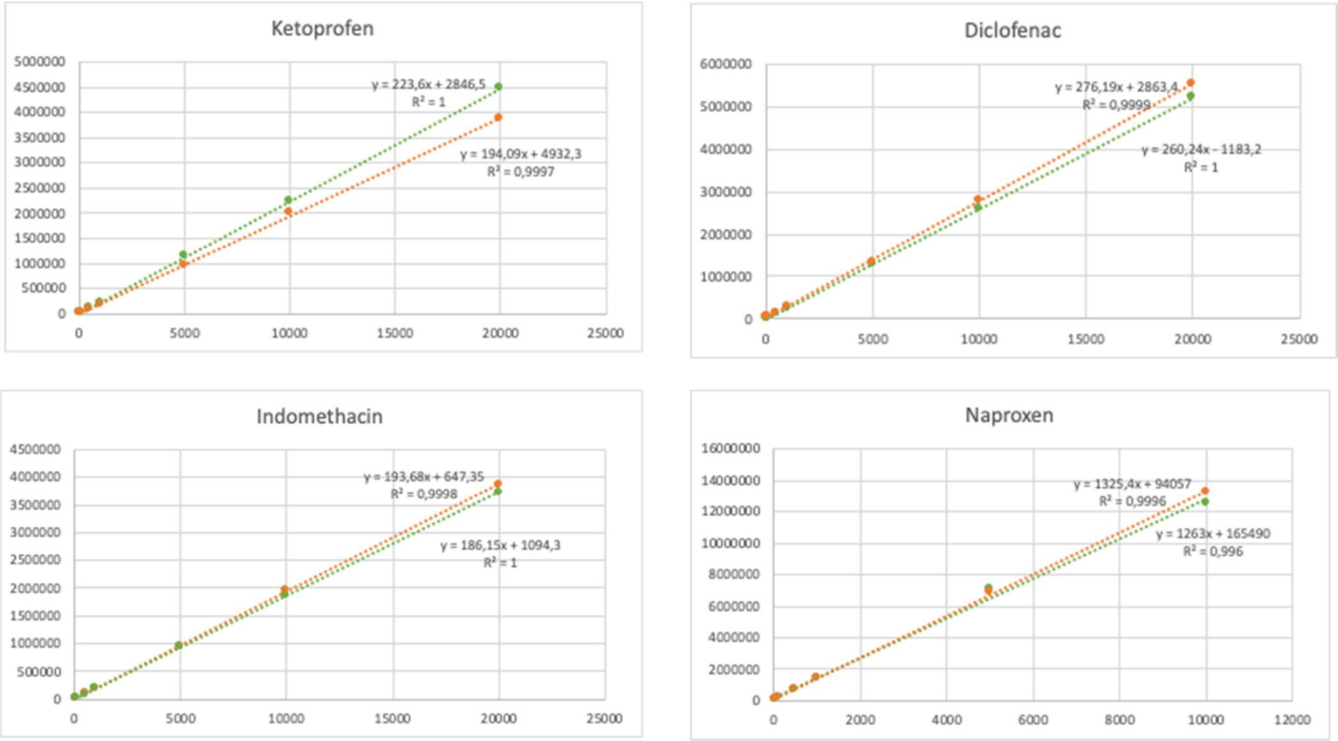

Figure S4. Calibration curves–matrix (green), standard (orange).
